# Spontaneous activity patterns in human motor cortex replay evoked activity patterns for hand movements

**DOI:** 10.1038/s41598-022-20866-5

**Published:** 2022-10-07

**Authors:** Tomer Livne, DoHyun Kim, Nicholas V. Metcalf, Lu Zhang, Lorenzo Pini, Gordon L. Shulman, Maurizio Corbetta

**Affiliations:** 1 Weizmann Institute of Science, 7610001 Rehovot, Israel; 2 Washington University in Saint Louis, St. Louis, 63110 USA; 3 Department of Neuroscience and Padova Neuroscience Center, University of Padova, 35131 Padova, Italy; 4 Venetian Institute of Molecular Medicine (VIMM), 35129 Padova, Italy

**Keywords:** Cognitive neuroscience, Motor control, Neural circuits, Sensorimotor processing

## Abstract

Spontaneous brain activity, measured with resting state fMRI (R-fMRI), is correlated among regions that are co-activated by behavioral tasks. It is unclear, however, whether spatial patterns of spontaneous activity within a cortical region correspond to spatial patterns of activity evoked by specific stimuli, actions, or mental states. The current study investigated the hypothesis that spontaneous activity in motor cortex represents motor patterns commonly occurring in daily life. To test this hypothesis 15 healthy participants were scanned while performing four different hand movements. Three movements (Grip, Extend, Pinch) were ecological involving grip and grasp hand movements; one control movement involving the rotation of the wrist was not ecological and infrequent (Shake). They were also scanned at rest before and after the execution of the motor tasks (resting-state scans). Using the task data, we identified movement-specific patterns in the primary motor cortex. These task-defined patterns were compared to resting-state patterns in the same motor region. We also performed a control analysis within the primary visual cortex. We found that spontaneous activity patterns in the primary motor cortex were more like task patterns for ecological than control movements. In contrast, there was no difference between ecological and control hand movements in the primary visual area. These findings provide evidence that spontaneous activity in human motor cortex forms fine-scale, patterned representations associated with behaviors that frequently occur in daily life.

## Introduction

Spontaneous cortical activity measured in a task free (resting) state is correlated between brain regions that are jointly recruited in different tasks and cognitive states^1–6^. Furthermore, the correlation between cortical regions at rest can be modified by learning, specifically in cortical regions recruited during novel motor, tactile, visual, or memory tasks^7–13^. These findings indicate that ongoing spontaneous activity in the resting state represent, at least in part, the global structure of behaviorally relevant brain processing. It is unclear, however, whether ongoing activity reflects mainly the communication between brain regions, i.e. fluctuations of activity between neuronal assemblies that through time-varying interactions facilitate or inhibit communication^14^, or whether ongoing activity also plays a representational role, i.e. coding or storing information about behaviorally relevant states^9,15–17^.

In humans, the spatial patterns of functional connectivity, i.e., the temporal correlation of the BOLD signal recorded with fMRI, at rest is more similar to that evoked by natural visual stimuli (e.g. movie segments) than synthetic stimuli (e.g. polar angle or eccentricity gratings)^16,18^. Finally, spontaneous patterns related to specific stimulus categories (faces, bodies, tools, places) occur more frequently in the appropriate category specific visual association area, i.e. face patterns in the fusiform face area, as compared to scrambled stimuli^17^. Overall, these results indicate a similarity between spontaneous (resting) activity and natural visually evoked patterns.

The daily repertoire of human hand movements can be decomposed into principal components^19^. Ingram et al. found two components that accounted for most of the variability of daily motor hand movements^19^. These and other results have suggested a theory of motor coding, referred to as motor synergies, which posits that the high dimensionality of arm and hand movements serving ecological and behaviorally useful functions are represented in the brain by a low dimensional set of activity patterns that code for co-occurring muscle and joint commands^20,21^. Leo et al. demonstrated in a neuroimaging study of human hand movements that activity in the motor cortex can be described in terms of motor synergies^22^.

**Figure 1:**
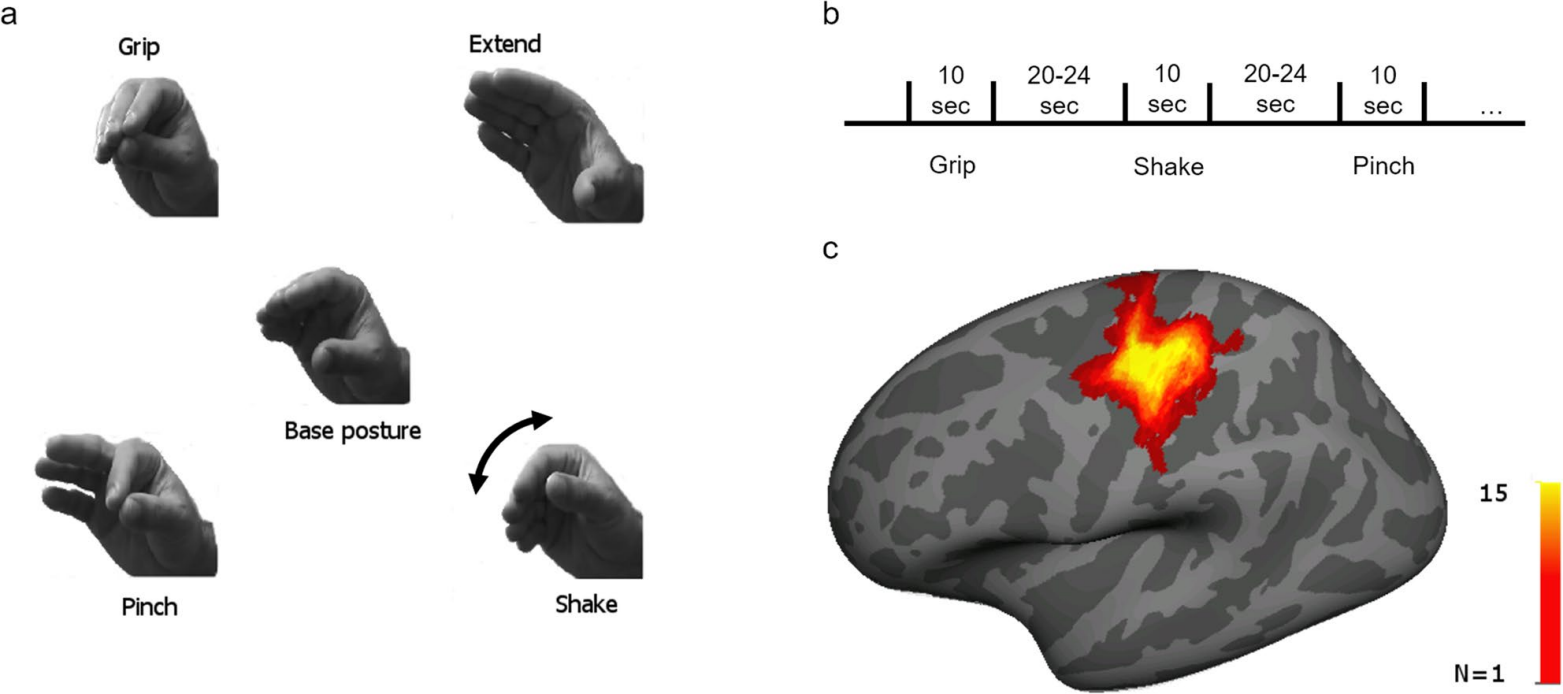
**(a)** The four different hand movements used in the study. Center—base hand posture for all movements. In each condition participants were asked to repeatedly and smoothly alternate between the base hand posture and one of the other four positions, creating the different hand movements. Top left—Grip movement, closing hand; top right—Extend movement, opening hand; bottom left—Pinch movement, closing only two fingers; bottom right—Shake movement, rotation of the wrist. Grip, Extend and Pinch were coded as ecological hand movements. Shake movement was chosen as the control movement. **(b)** experimental design—participants completed five task runs (except for one participant who completed seven runs, and another who completed only four), and each run included twelve motor blocks (three of each movement) of 10 s continuous hand movement. Task blocks were separated by rest blocks (20–24 s long). **(c)** Motor ROI—for each participant we identified a motor cortex region of interest (ROI) in which the BOLD activation for all the motor tasks was significantly higher than baseline activity. ROIs were identified on the native cortical surface of each participant. For presentation purposes only all ROIs were projected on an average surface (Freesurfer fsaverage) and summed together. The color scale represents the number of participants for which the specific vertex on the surface was included in the ROI. Talairach coordinates of the center of mass of the summed ROIs [− 36, − 23, 55].

As in visual cortex, we compare hand movements that are common and ecological with a less frequent control hand movement (Fig. 1a, Grip, Extend, Pinch vs. Shake). “Grip” and “Extend” correspond to the congruent finger extension and flexion that occur during a movement of hand release and grasp from a mid-opening position. Ingram et al. identified these two movements as the most commonly occurring in daily life^19^. The “Pinch” movement was included since it involves thumb-index opposition, the precision grip of the human hand. These ecological movements were contrasted (as in the case of natural vs. synthetic visual stimuli) with a control, unusual and not functional, movement involving the adduction/abduction of the flexed wrist. This “Shake” movement (Fig. 1a) is different not only in terms of frequency and ecological value, but it does involve different joints and direction of movement. Nonetheless it provides a suitable control as the main comparison is not among task-evoked patterns, but between resting state and movement patterns.

To test this hypothesis, we scanned healthy volunteers during resting state (fixation) fMRI scans immediately before and after movement scans. During movement scans (n = 5) they carried out in random orders blocks of the four movements performed with the right hand, alternating with fixation periods (Fig. 1b). Task-evoked patterns were computed for the movements to localize human motor cortex. Within this region we also computed the multivariate (multi-vertex as the analysis was carried out on the cortical surface) spatial pattern of activation in the four movements. Next, we compared frame-by-frame the similarity between resting state patterns (pre and post) and movement-evoked patterns in motor cortex. We found resting-state patterns to be more similar to the ecological hand movements evoked patterns than to the control movement patterns. These findings support a representational role of ongoing spontaneous activity in the motor system.

## Results

### Movement classification in the motor cortex

**Figure 2:**
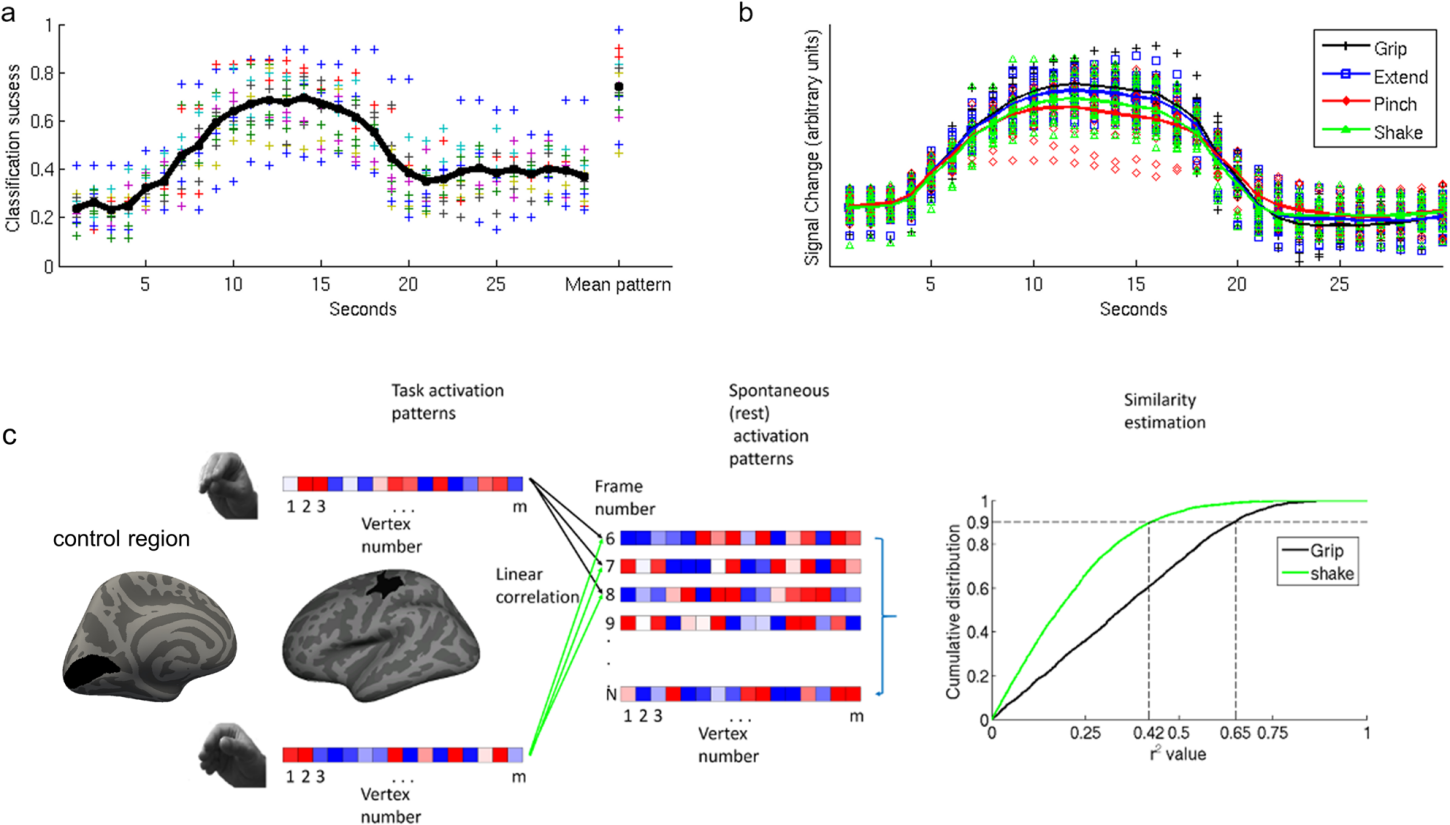
**(a)** Classification performance through a linear discriminant analysis indicated that the classification performance between the different hand movements through the pattern of motor activation at each TR followed the hemodynamic response function with a gradual increase in performance starting ~ 6 s after trial onset (spearman r between the mean classification function and the mean BOLD signal representing a full trial, combining all the conditions together, is 0.876). The rightmost data points represent classification performance based on the mean activation patterns constructed by averaging the patterns of the frames starting 10 s after trial onset and ending at 18 s after trial onset. Different colors represent different participants, the black trace represents mean classification levels across participants. Chance level of correct classification was 0.25. For presentation purposes only, each data point of the two participants who were scanned using a 2 s TR was duplicated in the figure (TR #1 is presented as second 1 & 2, etc.). **(b)** Mean BOLD time course for the four hand movement conditions. Each data point represents the mean normalized BOLD signal change of each participant to her baseline BOLD activity across all ROI vertices in a specific time point during the block. The solid lines represent the mean normalized BOLD signal change across participants corresponding to each hand movement. For presentation purpose only each data point of the two participants who were scanned using a 2 s TR was duplicated in the figure. **(c)** A spatial pattern was defined for each hand movement in the participant-defined motor ROI. Each of the four task patterns were compared to the spatial pattern of the same vertices in each rest frame using Pearson correlation. For each pattern, we computed the cumulative distribution function (CDF) of r^2^ values across all rest frames. In each CDF of each participant we calculated the value corresponding to the 90th percentile of that function (vertical dashed lines). This value was used to estimate the degree of representation of the specific pattern in the rest data. The same analysis was performed in a control ROI, the primary visual area.

**Figure 3:**
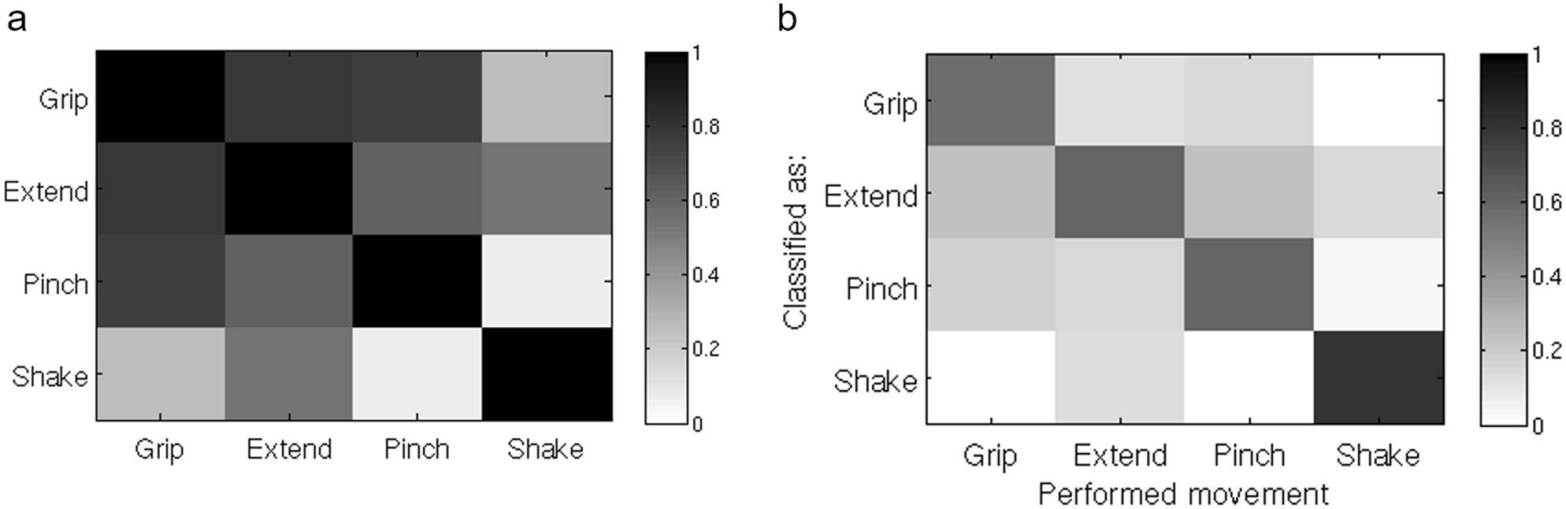
**(a)** Similarity matrix of task-defined patterns. Pearson correlation was used to estimate the similarity between the different patterns used in the main analysis. Similarity between the Grip, Extend, and Pinch patterns was higher than their similarity to the Shake pattern. the color scale represents the mean Pearson r values across all the participants. **(b)** Confusion matrix representing the mean correct classification rate of each movement pattern (diagonal) and the misclassification rate as a function of the incorrect response given by the classifier. The figure shows that the similar patterns (Grip, Extend, and Pinch) were misclassified as each other more often than they were misclassified as the Shake movement. The color scale represents the fraction of cases in which the tested pattern (x-axis—the performed movement) was classified as each of the four movements (y-axis).

To verify that task-evoked BOLD patterns within the motor ROI (Fig. 1c) were associated with specific hand movements, we tested whether the different movements could be discriminated using linear discriminant analysis (LDA). Using a leave-run-out cross-validation approach, we calculated classification success for the motor ROI in each acquired brain volume (1 volume per TR) within each block. Classification was significantly better than chance level (see Fig. 2a; *p* < 0.001, FDR-corrected, for the frames starting 10 s after trial onset and ending 8 s later, chance level 25%). The time-course of the classification success-rate function was highly correlated with the time-course of the mean BOLD signal (Fig. 2b; Spearman r^2^ = 0.77). Since our main aim was to compare task patterns to spontaneously emerging BOLD patterns, we constructed a mean activation pattern for each movement by averaging the patterns during the most activated TRs in a block (the period 10–18 s after the beginning of the block). Classification success rates using these mean patterns were high and significantly above chance (t(14) = 14.23, *p* < 0.001, mean classification success 0.73, se = 0.033—rightmost column in Fig. 2a). A representational similarity analysis (Fig. 3a), in which the patterns for the different movements were spatially correlated, indicated that the Grip, Extend, and Pinch patterns were more like each other than to the control (Shake) pattern. The high similarity between these three patterns was also apparent in the confusion matrix of the classifier (Fig. 3b). Misclassification tended to be between these three movements and the classifiers rarely misclassified one of these three movements as the Shake movement.

### Spontaneous occurrence of movement patterns during rest in human motor cortex

**Figure 4:**
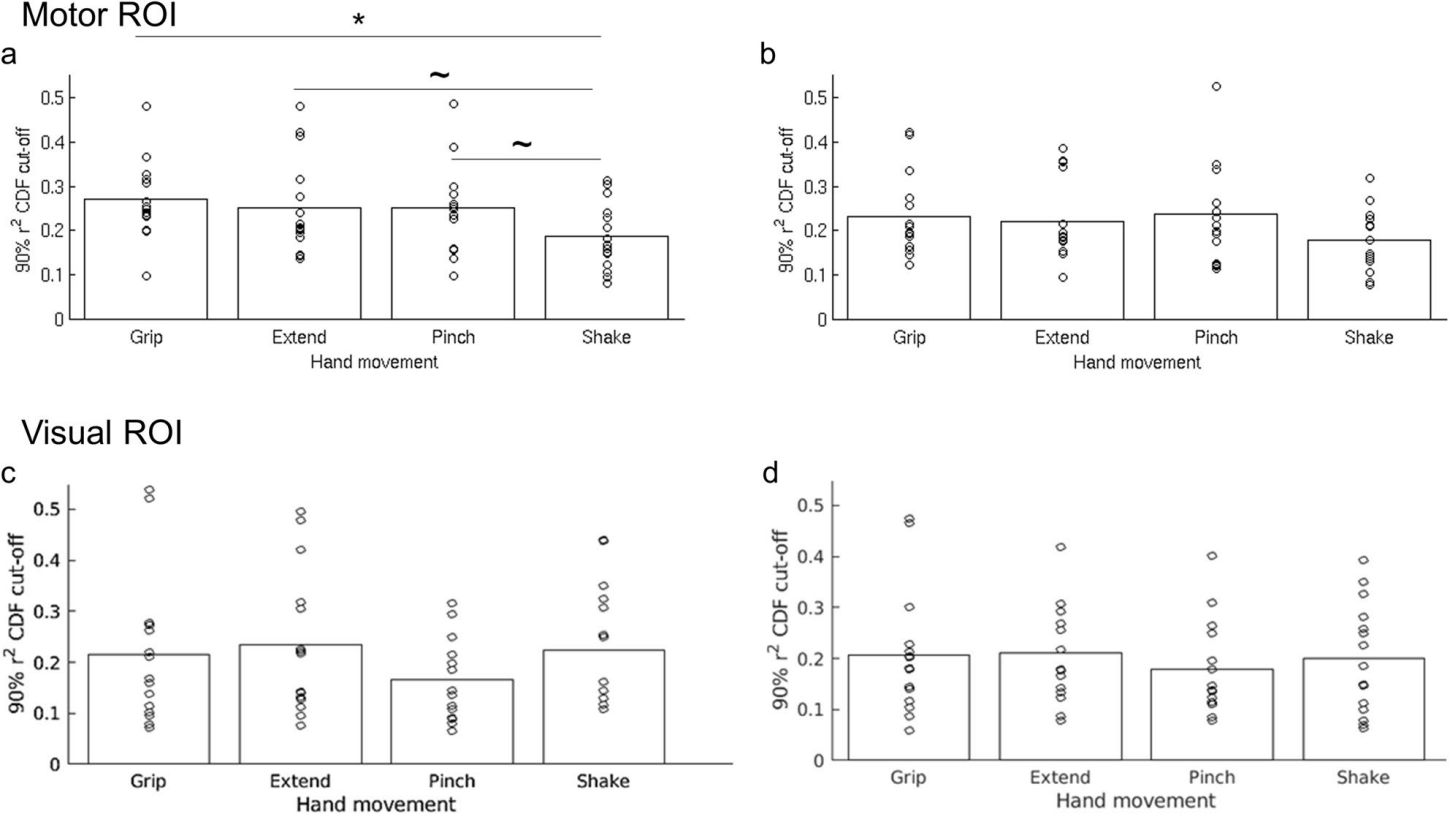
**(a)** r^2^ cut-off values of the four different hand movements’ patterns in the pre-task rest data (all participants). Grip had the highest cut-off value indicating that this pattern was most represented in the pre-task spontaneous activity. The lowest cut-off value was that of the Shake condition, indicating the it was the least represented pattern in the pre-task resting data. The difference between the Grip and the Shake conditions was significant following a correction for multiple comparisons (*). Both comparisons—Extend vs Shake, and Pinch vs Shake were significant before correction, but did not survive the correction for multiple comparisons ( ~). **(b)** r^2^ cut-off values in the post-task rest data. No significant differences were found in the post-task data in terms of r^2^ cut-off values of the different conditions. Bottom panels: r^2^ cut-off values in the pre-task rest **(c)** and post-task rest **(d)** data in the visual area. No significant differences were found in both timepoints in terms of r^2^ cut-off values of the different conditions.

To test whether hand movements are represented in spontaneous activity, the mean task patterns were compared to each pre-task resting state frame using Pearson correlation. An overall similarity measure between the task pattern and spontaneous rest patterns was defined as the 90^th^ percentile value of the cumulative distribution function of the r^2^ values of each pattern (see Fig. 2c). With this method of quantifying similarity, a higher cut-off value indicates that a larger fraction of rest frames had a higher similarity to the tested pattern than to a pattern having a lower cut-off value. We conducted a 1-way repeated measure ANOVA with pattern as the independent variable, 90^th^ percentile cut-off value as the dependent variable, and participant as a random variable of no concern. The analysis indicated a main effect of movement type (F(3,42) = 5.46, *p* = 0.003). Results are presented in Fig. 4a. Post hoc paired-sample t-tests indicated that the rest patterns were significantly more like the Grip than Shake patterns (t(14) = 3.49, *p* = 0.004,r^2 ^cut-off = 0.272 and 0.186, respectively), an effect that remained significant after correcting for multiple comparisons using the Holm-Bonferroni sequential method. The Extend and Pinch patterns’ cut-off values (0.252 and 0.25, respectively) were also higher than the Shake cut-off value but these differences did not survive multiple comparisons (t(14) = 2.34, *p* = 0.034, and t(14) = 2.35, *p* = 0.034, respectively). These results support our hypothesis that ecological hand movements are more frequently represented in the pattern of resting, spontaneous activity in motor cortex, compared to a control hand movement.

### Reduction in spontaneous representation of movement patterns following task performance

**Figure 5:**
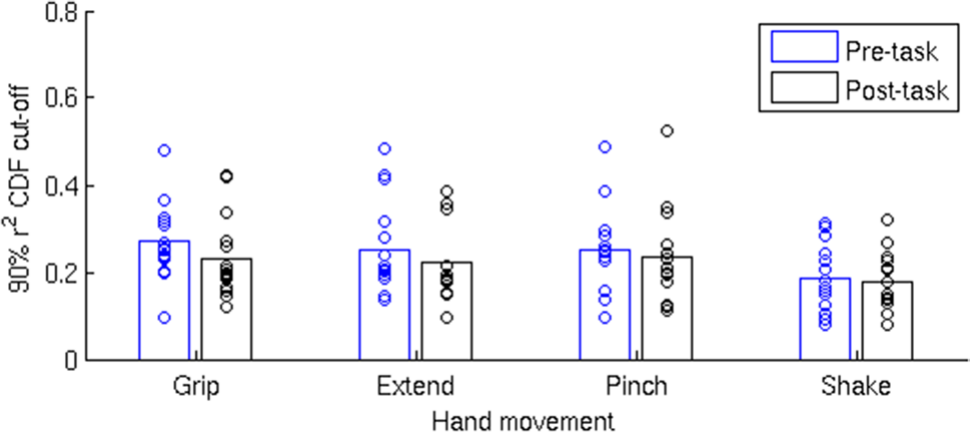
A comparison between the similarity of each task-defined pattern and the pre- and post-task rest spontaneous patterns. In the Grip and the Extend condition there was a trend indicating a reduction in the overall observed similarity in the post-task rest relative to the pre-task rest. No such reduction was observed for the Pinch and Shake conditions.

When the same analysis was conducted on the post-task rest frames, the main effect of movement type was no longer significant (F(3,42) = 2.43, *p* = 0.078, Fig. 4b). This null result was largely due to a trend indicating a reduction in the similarity between the Grip and rest patterns (post-task *vs* pre-task: t(14) = 2.79, *p* = 0.014, non-corrected), as well as between the Extend and rest patterns (post-task *vs* pre-task: t(14) = 2.42, *p* = 0.030, non-corrected), coupled with the lack of any reduction in the similarity between the Pinch and Shake conditions and rest patterns (both *p* > 0.3) (Fig. 5).

### Control analysis in the left visual cortex

The above analysis was repeated in a control visual ROI. Specifically, V1 was selected according to the assumption that these neurons process visual inputs and are selective for different attributes, including direction of motion, orientation, spatial and temporal frequency^23^. By means of this analysis we controlled whether an early distinction between ecological *vs* control patterns would be observed during the visual processing stage, driven by possible different attributes of the four hand movements. Results showed no significant differences in V1 among movements in their task-rest similarity (Fig. 4c,d) (pre-task: F (3,42) = 1.31, *p* = 0.28; post-task: F (3,42) = 0.35, *p* = 0.79; pre- and post-task: movement type: F (3,42) = 0.80, *p* = 0.50; time: F (1,42) = 1.33, *p* = 0.27; movement type x time: F (3,42) = 1.79, *p* = 0.16). This analysis suggested that the results in the motor ROI are independent of visual input processing.

### Spatio-temporal analysis

**Figure 6:**
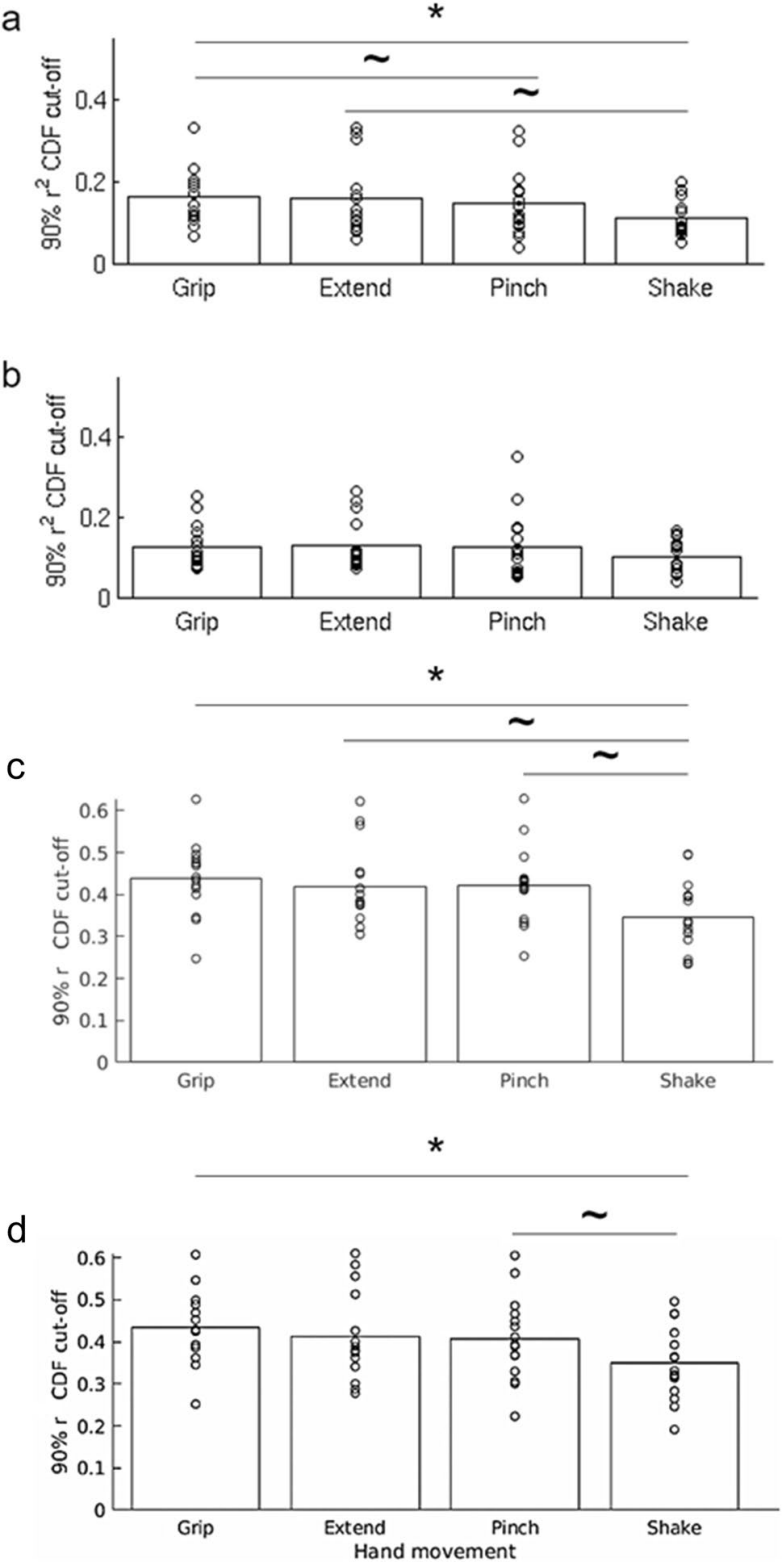
**(a)** r^2^ cut-off values of the four different hand movements’ spatio-temporal patterns in the pre-task rest data. There was a significant main effect of movement (F(3,42) = 5.36, *p* = 0.0032) in a repeated measures ANOVA. Grip had the highest cut-off value (0.165) indicating that this pattern was the one most represented in the pre-task spontaneous activity. The lowest cut-off value was that of the Shake condition (0.11), indicating that it was the one least represented in the pre-task data. Only the difference between the Grip and the Shake conditions was significant following a correction for multiple comparisons (* t(14) = 3.66, *p* = 0.0026). The comparisons of Extend (0.159) vs Shake and Grip vs Pinch (0.145) were significant before correction but did not survive correcting for multiple comparisons (~ t(14) = 2.485, *p* = 0.026, and t(14) = 2.21, *p* = 0.044, respectively). **(b)** r^2^ cut-off values in the post-task rest data using the spatio-temporal patterns. No significant differences were found in the post task data in terms of r^2^ values of the different conditions. **(c)** The main analysis was repeated considering r values (instead of r^2^ values) for the computation of the 90th cut-off of the CDF, and **(d)** inverting r values (multiplied by -1) – refer to the text for details.

The spatio-temporal analysis tests for the similarity of the pattern dynamics between movement and rest, essentially looking at similarities over the time window of a motor activation (8 s). The pre-task rest data showed a main effect of movement type (F(3,42) = 5.36, *p* = 0.003) (Fig. 6a). Post-hoc paired-sample t-tests indicated that the Grip pattern was significantly more similar to the rest patterns than the control (Shake) pattern (t(14) = 3.66, *p* = 0.003) after correcting for multiple comparisons. The Extend pattern’s cut-off values were also higher than the Shake value but this difference did not survive multiple comparisons (t(14) = 2.48, *p* = 0.026). The Grip and Pinch conditions were also different (t(14) = 2.21, *p* = 0.044), but did not survive correction. When the same analysis was conducted on the post-task rest frames (Fig. 6b) the main effect of the movement type was no longer significant (F(3,42) = 1.23, *p* = 0.31). Additionally, as in the main analysis, the changes between the pre- and post-task rest similarity with the task patterns resulted from decreased similarity of the post-task rest to the Grip (t(14) = 3.28, *p* = 0.006, significant after correction) and to the Extend (t(14) = 2.57, *p *= 0.022, approaching significance) conditions relative to the pre-task rest.

### Pattern similarity analysis using Pearson r values

Finally, we considered the whole spectrum of r values (compared to r-squared in the main analysis) to compute the 90th CDF cut-off (thus concentrating on the highest nominal correlation values). We confirm the main results (Fig. 6c), showing a significant main effect of movement type (F(3,42) = 6.19, *p* = 0.001). Post-hoc paired-sample t-tests indicated that the rest patterns were more different from the control than the ecological gestures (Shake vs Grip: t(14) = 3.59, *p* = 0.003, significant after correction; Shake vs Extend: t(14) = 2.54, *p* = 0.024, not significant after correction; Shake vs Pinch: t(14) = 2.76, *p* = 0.016, not significant after correction). When we inverted the distribution (multiplying correlation values by -1), we still found a significant main effect of movement type (F(3,42) = 4.61, *p* = 0.007), similar to the above analysis (Shake vs Grip: t(14) = 3.20, *p* = 0.006, significant after correction; Shake vs Pinch: t(14) = 1.84, *p* = 0.041, not significant after correction) (Fig. 6d). These analyses point out to the fact that the overall rest patterns’ variability is indeed best accounted for by the ecological hand movements, exhibiting both higher-positive and higher-negative correlation with those movements than with the control movement.

## Discussion

In the current study, we tested the hypothesis that ongoing cortical spontaneous activity contains patterns of activity related to behavioral states that occur frequently in daily life ^15^. We tested this hypothesis in the human primary motor cortex using ecological hand movements that were compared to an infrequent and non-ecological hand movement. In agreement with previous research, we found that it is possible to decode different hand movements in human motor cortex using fMRI^22,24–27^. Interestingly, the spatial pattern of activation for ecological movements (grip, extend, pinch) were more like each other than the control (Shake) movement (Fig. 3a). Notably, the mean (variance) of the motor response was similar across all four movements and comparable across subjects hence unlikely to drive the classification (Fig. 2a, b).

Next, we tested whether movement activation patterns in motor cortex were present in resting state activity, and whether they occurred more frequently for the ecological movements. To avoid short-term adaptation or motor learning, we used resting state activity recorded prior to the motor tasks. At that time subjects had received a brief and equal exposure to each of the hand movements during the instruction period prior to scanning. The results show that resting state patterns in human motor cortex were more like activation patterns for ecological movements than for the shake movement (Fig. 4a). The measure of similarity was the 90% point of the CDF distribution of Pearson r^2^ values (considering both positive and negative r values) between task and rest scan patterns in motor cortex. The higher this value the more frequently patterns at rest resemble task patterns and account for their overall variability patterns. A statistical difference was detected only for the Grip vs. Shake, but the pattern of results suggests that all three ecological movements were more frequently represented at rest than the control Shake movement. The r^2^ cut-off for Grip = 0.272; Extend = 0.252; Pinch = 0.25 were all very similar as compared to the r^2^ cut-off for Shake = 0.186 (Fig. 4). This is also consistent with their similarity in terms of task activation patterns (Fig. 3).

It is important to highlight that we are measuring a change of the variance of the distribution of r values, not their mean. In other words, when looking at the extreme right end of the distribution (90^th^ percentile point) ecological movement patterns account significantly more than the shake pattern for the observed resting state pattens. Since the distribution of r values is computed frame-by-frame across 15 min of resting state scans, it means that resting state patterns match more frequently ecological movement patterns compared to the control gesture. Critically, this finding was confirmed when looking only at the positive r values, i.e., a voxel with high signal during task corresponds to a voxel with high signal during rest. When looking at r^2^ both positive and negative correlations between spatial patterns are considered. This result (higher variance similarity) matches perfectly recent findings in human visual cortex for category specific object stimuli ^17^.

Differences in rest-task similarity patterns between hand movements were not detected in area V1. Since subjects could not look at their hand during the movements, even though they received somatosensory feedback, we selected area V1 as a control area in which familiarity with the movement was not expected to have an effect. We found in V1 that the 90th percentile r^2^ value was lower on average than in motor cortex and did not show a movement frequency effect (Fig. 4c). While we cannot say based on this analysis whether the levels of task-rest similarity in V1 reflects just noise, under different conditions spontaneous activity in V1 can reflect task patterns. In Kim et al. we found that resting state activity in V1-V2 was more likely to resemble phase scrambled visual objects than real objects^17^. What controls spontaneous vs. task-driven activity in the cortex remains a fascinating topic for future work.

When the same analysis was conducted using resting state scans that were collected immediately following task performance, the difference between the conditions was no longer significant. The representation of the hand movements that were most present in the pre-task resting activity (grip, extend) (Fig. 5) was diminished while the other hand movements were relatively unchanged. This selective pattern of changes suggests that the lack of significant effect in the post-task comparison is not due to an overall reduction in neural activity or a reduction of the overall variability of neural activation in the ROI, but instead reflected a long-term (time scale of minutes) pattern-specific adaptation process similar to the one commonly observed online during task performance^28,29^. Such long-term adaptation of spatial patterns in visual cortex has been previously reported in the resting state following learning to perform a novel visual discrimination task^30^. The adaptation for the most common movements at rest is also consistent with recent evidence indicating that decrements of task activity for familiar (not novel) motor sequences are the hallmark of motor learning in fMRI^31^.

Similar results to those obtained with the mean pattern were obtained also using a spatio-temporal pattern in which the pattern across the chosen TRs was concatenated to one another and compared to similarly concatenated resting-state frames. This result suggests that any non-stationarity^32–37^ in the fMRI resting-state spontaneously emerging patterns is modulated by slow transitions, which is consistent with the known temporal properties of the hemodynamic response function estimated using task data^38–41^. Alternatively, it might suggest that the spontaneous activity represents a weighted combination of all the represented patterns, in which case the most dominant one across most TRs would be the grip pattern. Further studies will be required to determine which option is correct.

An open question that will require further research is how the motor cortex dissociates between spontaneous patterns that do not require the initiation of an actual motor act and patterns involving an actual demand for real movement. One possibility is that of gating by other regions (premotor, thalamus). Such a mechanism would suggest that every motor plan is composed of at least two components, an instruction for action and an instruction for a control area, to allow the motor plan to pass down the efferent pathway. Some support for this suggestion is found by comparing motor execution to motor imagery in healthy participants^42^ and in amputees^43^. These studies reported a difference between motor imagery and motor execution activation in the recruitment of the supplementary motor area. Another option is a threshold-based mechanism that requires a high match between the activated pattern and the optimal pattern for that specific motor action. This may involve the motor cortex with other functionally connected regions. A fascinating question is whether movement-like patterns in motor cortex may be synchronized with similar movement-like patterns in other regions. In Kim et al. we found that object-specific patterns (e.g., faces) were temporally synchronized across multiple visual areas coding for the same stimulus^17^. We defined this form of interaction ‘pattern connectivity’. In future work we plan to look at the interaction of movement patterns in motor cortex with other regions more or less functionally interacting both at rest and during tasks.

This experiment has several limitations. We did not measure the kinetics of hand movements in the scanner, but only observed their execution. Aside from the objective technical challenges, this limitation is attenuated by the covariance pattern of activation clearly indicating an orderly spatial pattern of activation between more common and novel movements. The similarity in response magnitude and variance across subjects indicated a good homogeneity in task performance.

A second limitation is that we did not explore many hand movements or postures, so the conclusion that the statistics of movement in everyday life is a determining factor in shaping resting state activity is indirect and is based on previous work^19^. Similarly, further studies should assess whether these results are confirmed using different hand gestures relying on a familiarity/novelty gradient.

Finally, the number of subjects was relatively small, and this factor may have prevented to obtain statistically different results for all ecological movements as compared to the control movement.

Overall, previous work has indicated that ongoing spontaneous cortical activity can shape and even be used to predict success in a variety of perceptual and cognitive tasks^44–50^ as well as account for behavioral deficits in patient populations^51,52^. The current finding that spontaneous activity represents behaviorally meaningful information can help explain how internal representations that incorporate statistical regularities in the environment and behavior might shape the automatic interpretation and response to events in our daily life. Specifically, we have proposed that spontaneous brain dynamics during resting periods optimize generative models for future interactions by maximizing the entropy of explanations in the absence of specific data^15^.

## Methods

### Experimental Design

Participants (N = 15, 7 females, mean age 26.5, all right-handed) performed a block-design hand-movement task in which they were instructed by a visual word cue presented on a mirror-set inside the magnet, to move their right hand repeatedly for 10 s in one of four movements followed by a variable rest period (20–24 s), see Fig. 1. The sample size for this study was estimated based on previous studies comparing patterns of spontaneous neural activity to patterns evoked by stimuli^7,8,16^. Moreover, this sample size is largely overlapping with our previous study investigating the spontaneous representational role in the visual cortex^17^.

Four hand movements were used in the current study: Grip, Extend, Pinch, and Shake (Fig. 1a). Grip and Extend match the most common motor synergies and correspond to the first two principal components identified in the work of Ingram et al.^19^. The Pinch movement involves thumb-index opposition and represent the precision grip movement of the human hand. The Shake hand movement involves the adduction/abduction movement of the flexed wrist. This movement is not frequent and not ecological.

To prevent movement artifacts in the scanner due to the hand movements during task performance the right arm was placed inside a padded plastic half pipe and strapped to it. There were five task runs (except for one participant who performed seven runs and another that performed only four runs), in each run each movement occurred 3 times in a random order. The same participants were also scanned in three five-minutes pre-task resting state scans and three five-minutes post-task resting state scans (except for one participant who only performed 2 post-task resting state scans) to evaluate the spontaneous occurrence of the BOLD patterns associated with the different hand movements during rest, and the effect of task performance on the spontaneous activity. During rest scans the participants kept their arm in the restraining mechanism and were instructed to rest their palm on the scanner bed alongside their body. Task performance was verified visually throughout the study. All participants signed an informed consent form and the study was approved by Washington University IRB. All methods used in the current study were performed in accordance with the relevant guidelines and regulations of the ethical review board.

The experiment was controlled using the Psychtoolbox extension to Matlab^53,54^. Stimuli were presented to the participants using a head mounted mirror set and a back projector.

### MRI acquisition

The data for 2 participants were acquired using a Siemens (Erlingen, Germany) 3-T Tim Trio MR system, using a 16-channel RF head coil. Whole-brain single-shot echo-planar images were acquired using the following parameters: 32 interleaved slices, 2-s repetition time (TR), 27-ms echo time, 4-mm thickness. Voxel size was 4 × 4 × 4 mm. Up to two high-resolution T1-weighted echo-planar MPRAGE anatomical images (isotropic 1-mmvoxels) were obtained and used to construct a high-resolution image of each participant’s brain.

The data for the rest of the participants were acquired using a Siemens (Erlingen, Germany) 3-T Prisma Fit MR system, using a 32-channel RF head coil. Whole-brain echo-planar images with multiband factor of 3 were acquired using the following parameters: 56 interleaved slices, 1-s repetition time, 25.8-ms echo time, 3-mm thickness. Voxel size was 3 × 3 × 3. Up to two high-resolution T1-weighted multi-echo MPRAGE anatomical images (isotropic 1-mmvoxels) were obtained and used to construct a high-resolution image of each participant’s brain.

Structural and functional preprocessing were performed using the FreeSurfer and FS-FAST processing stream developed at the Martinos Center for Biomedical Imaging (surfer.nmr.mgh.harvard.edu). Frames were motion-corrected by aligning them to the middle frame in each run. Functional data were registered to the high-resolution structural data and then segmented into left and right cortical surfaces. The intensity level of each frame was normalized. The raw time series was then resampled to the reconstructed surfaces. A 5-mm FWHM Gaussian smoothing was applied to the resampled data. The frames corresponding to the first four TRs of each scan were discarded from the analysis.

### Statistical analysis

We first defined a motor region of interest (ROI) by performing a general linear model analysis contrasting motor performance with baseline activity (four movements combined to avoid biasing the ROI selection to a specific movement), using a gamma function defined by the following parameters: Δ = 2.25 and τ = 1.25. Motion correction parameters were used as nuisance regressors in this analysis. We then defined a central-sulcus cluster on the native surface of each participant in which the contrast was significant. To keep the size of the ROIs similar across participants we adjusted the p-level of the contrast until we ended up with an ROI size of 2187 vertices ± 7%.

For the main analyses we additionally regressed out the global (mean) signal of the left hemisphere from the temporal activity pattern of each vertex. We then normalized the activity levels in each TR by converting them to z-score values ending up with a relative activation pattern of the different vertices in each TR.

We tested the discriminability of the cortical activation patterns induced by the different hand movements in the motor ROI using a linear discriminant analysis (LDA) performed in Matlab (Mathworks inc). Classification success was estimated using a cross-validation leave one run out method.

A mean pattern for each hand movement (constructed from all the task runs) was compared to each resting state frame using Pearson correlation. We computed the cumulative distribution function (CDF) of the r^2^ values and identified the r^2^ value that represented the cumulative 90% point. The higher this value is the more of the activity pattern during rest is explained by the task pattern. Additionally, we compared the representation of each movement condition in the pre- and post-task rest spontaneous activity.

All t-tests reported were two-tailed and paired-sample when appropriate. The Holm-Bonferroni sequential method of correction for multiple comparison was used when appropriate for the main analyses. An FDR correction for multiple comparison was performed in the classification analysis. All reported p-values are uncorrected. All ANOVA test comparing effects within subjects were conducted as repeated-measures ANOVA.

We also identified a control ROI, primary visual area (i.e., Brodmann area 17, V1) in the left hemisphere, which was computed from Brodmann Areas atlas included in FreeSurfer (https://surfer.nmr.mgh.harvard.edu/fswiki/BrodmannAreaMaps)^55^ For each subject, the Freesurfer V1 label was registered to the native space through the inverse matrix of the fsaverage transformation. The corresponding native-registered V1 ROI was used for this control analysis, performing the same procedure used in the main analysis.

We conducted two supplementary analyses to further assess the nature of the main effect. We repeated the main analysis using spatio-temporal pattern to investigate the temporal dynamics of the activity patterns evaluated in the main analysis. For this analysis the patterns across the chosen TRs were concatenated to one another, creating an eight second cortical activation matrix (vertex × TR), and compared to similarly concatenated consecutive resting-state frames using Pearson correlation (each of the two matrices was converted to a 1-dimensional vector for this purpose).

In the second supplementary analysis, the cumulative 90% cut-off of the CDF distribution used to assess similarity between patterns of movements and rest was computed using Pearson correlation r values (instead of the r^2^ values used in the main analysis). This analysis was conducted to evaluate the contribution of positive correlation values to the effect identified in the main analysis, following the assumption that positive values indicate a positive match between task and rest patterns and have a more straightforward interpretation. Similarly, we repeated this analysis inverting the nominal value of r-correlations (that is, multiplying r values by − 1, thus looking at the 90% cut-off of the inverted distributions—which is equivalent to looking at the 10% cut-off of the original distribution).

## Data availability

The dataset analyzed in this study is stored at cnda.wustl.edu; Restrictions apply to the availability of the dataset which was analyzed in the current study under license from the cnda center in Washington University in St. Louis. The Data-set in not publicly available, however it could be made available upon reasonable usage request from TL and MC along with the permission of the cnda center (cnda.wustl.edu).
